# Cryptotanshinone inhibits IgE-mediated degranulation through inhibition of spleen tyrosine kinase and tyrosine-protein kinase phosphorylation in mast cells

**DOI:** 10.3892/mmr.2021.12283

**Published:** 2021-07-12

**Authors:** Sumiyasuren Buyanravjikh, Sora Han, Sunyi Lee, Ae Lee Jeong, Hye In Ka, Ji Young Park, Ariundavaa Boldbaatar, Jong-Seok Lim, Myeong-Sok Lee, Young Yang

**Affiliations:** Department of Biological Science, Sookmyung Women's University, Seoul 04310, Republic of Korea

Mol Med Rep 18: 1095-1193, 2018; DOI: 10.3892/mmr.2018.9042

Following the publication of the above article, an interested reader drew to the authors’ attention that they had mentioned that activated PKCδ phosphorylates IKKβ in order that IKKβ is relocated to the plasma membrane, resulting in the induction of mast cell degranulation; however, four references the authors had included did not seem to support this statement. The authors have re-examined their paper, and realized that the four references the reader mentioned were indeed cited incorrectly, and wish to rectify this error through revising the third paragraph in the Discussion section, the References section, and an associated figure (Fig. 6C) in order to avoid any further misunderstandings on the part of the readership.

First, the authors wish to revise the wording of the third and fourth paragraphs of the Discussion, as featured on pp. 1101-1102, to the following (changed text is indicated in bold):

‘We showed that CRT exerts anti-AD effect through inhibition of the mast cell degranulation in mast cells. Upon IgE/antigen stimulation, the immunoreceptor tyrosine-based activation motif (ITAM) region of FcεRI receptor which is on the mast cell surface is phosphorylated and the initial signalling protein kinases Lyn and Syk are recruited to the ITAM **(28,29). Then, the activated** Lyn and Syk leads to phosphorylation of the transmembrane adaptor linker for activation of T cells (LAT). Phosphorylated LAT which is a scaffold for multimolecular signalling complexes and activates PLCγ through phosphorylation. The activated PLCγ hydrolyses phosphatidylinositol biphosphate (PIP2) to generate second signalling molecules IP3 and DAG, **which activate PKCs including PKCδ to induce the mast cell degranulation (30,31). On the other hand, cross-linking of** FcεRI also activates IKKβ, **which moves to the lipid raft fractions and phosphorylates synaptosomal-associated protein 23 (SNAP-23) leading to degranulation (7). Since PKCδ phosphorylates IKKα, but not IKKβ (32), it is not likely that two signalling pathways are directly connected.** In this study, novel function of CRT on phosphorylations of Lyn/Syk kinases in mast cells is elucidated for the first time. Furthermore, it is likely that this inhibitory effect of CRT on Lyn/Syk kinases negatively affected activities of their downstream signalling molecules including PLCγ, PKCδ, and IKKβ, which leads to decrease in mast cell degranulation by CRT treatment.

Besides the inhibitory effect of CRT on mast cell degranulation, here we provide additional evidence that CRT exerts anti-AD effects through inactivation of MAPK and NF-κB. It has been reported that CRT regulates the activities of MAPK and NF-κB in various cell types. In rhabdomyosarcoma, hepatoma, and breast carcinoma, CRT activates MAPK p38/JNK and suppresses ERK1/2, followed by caspase-independent apoptosis (10,**33,34**). In chronic myeloid leukaemia cells, CRT enhances TNF-α-induced apoptosis through the activation of MAPK p38 (**35**). In smooth muscle cells, CRT exerts anti-migration/invasion effect as it inhibits TNF-α/NF-κB signalling pathway (**36**).’

Secondly, the authors wish to make the following changes to the Reference list: New references 30–32 have been inserted to the list, as follows:

30. Ozawa K, Szallasi Z, Kazanietz MG, Blumberg PM, Mischak H, Mushinski JF and Beaven MA: Ca^2+^-dependent and Ca^2+^-independent isozymes of protein kinase C mediate exocytosis in antigen-stimulated rat basophilic RBL-2H3 cell. J Biol Chem 268: 1749–1756, 1993.

31. Cho SH, Woo CH, Yoon SB and Kim JH: Protein kinase Cδ functions downstream of Ca^2+^ mobilization in FcεRI signaling to degranulation in mast cells. J Allergy Clin Immunol 114: 1085–1092, 2004.

32. Yamaguchi T, Miki Y and Yoshida K: Protein kinase Cδ activates IκB-kinase α to induce the p53 tumor suppressor in response to oxidative stress. Cell Signal 19: 2088–2097, 2007.

The addition of these new references means that the former references 30–33 have been accordingly renumbered to references 33–36.

Finally, the authors have revised Fig. 6C, as it appeared on p. 1102, in order to assist the understanding of the readers, and the corrected version of Fig. 6 appears on the next page. All these corrections have been approved by all the authors, with the exception of the first author, Sumiyasuren Buyanravjikh, who is no longer uncontactable. The authors regret that these errors were included in the paper, even though they did not substantially alter any of the major conclusions reported in the study, are grateful to the Editor for allowing them this opportunity to publish a Corrigendum, and apologize to the readership for any inconvenience caused.

## Figures and Tables

**Figure 6.**
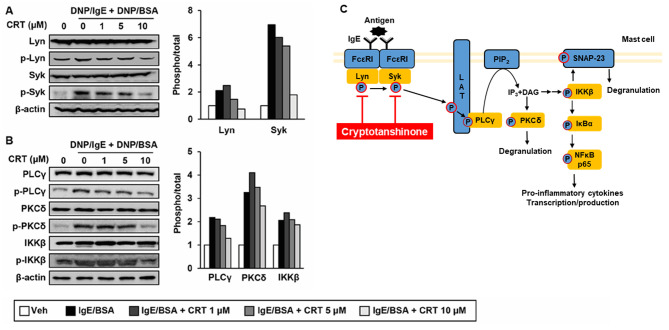
CRT inhibits the signalling pathways of Lyn and Syk (A and B) RBL-2H3 cells were sensitised with anti-DNP-IgE for 16 h followed by treatment with various concentrations of CRT 30 min before DNP/BSA stimulation. Levels of phosphorylated (A) Lyn and Syk, and (B) PLCγ, PKCδ and IKKβ were determined by western blot analysis 1 h after DNP/BSA stimulation. The relative ratio of phosphorylated protein to total protein levels were quantified. (C) Schematic diagram indicates how CRT suppresses mast cell degranulation. CRT, cryptotanshinone; DNP/IgE, anti-dinitrophenyl IgE isotype; DNA/BSA, dinitrophenyl-bovine serum albumin; PLCγ, phospholipase Cγ; PKC, phospho-protein kinase C; IKK, IκB kinase; IgE, immunoglobulin E; Veh, vehicle; Syk, spleen tyrosine kinase.

